# Simplified Protocol for the Purification of Native Cas Nucleases for DNA-Free Genome Editing

**DOI:** 10.3390/mps8010016

**Published:** 2025-02-07

**Authors:** Margherita D’Amico, Flavia Angela Maria Maggiolini, Lucia Rosaria Forleo, Maria Francesca Cardone, Riccardo Velasco, Teodora Basile, Carlo Bergamini

**Affiliations:** CREA Council for Agricultural Research and Economics—Research Center for Viticulture and Enology, Via Casamassima 148, 70010 Turi, Italy; flavia.maggiolini@crea.gov.it (F.A.M.M.); luciarosaria.forleo@crea.gov.it (L.R.F.); mariafrancesca.cardone@crea.gov.it (M.F.C.); riccardo.velasco@crea.gov.it (R.V.); teodora.basile@crea.gov.it (T.B.)

**Keywords:** Cas purification, ribonucleoprotein, genome editing, affinity chromatography, ion exchange chromatography

## Abstract

DNA-free genome editing by the direct delivery of CRISPR-associated nucleases has emerged as a promising technology due to its precision and reduced risk of off-target effects. However, existing purification protocols for native Cas proteins require the use of complex instrumentation, which limits their application. Here, we present a simplified protocol for the purification of native Cas9, Cas12RR and dCas9-VP64 nucleases optimized for DNA-free genome editing. Our approach leverages a streamlined affinity and ion exchange chromatography coupled with minimal downstream processing, ensuring a good yield and activity of the purified proteins. The in vitro analysis of the purified ribonucleoprotein complex demonstrated a good efficiency of DNA target cleavage. This simplified protocol increases the opportunity to adopt CRISPR technology, and enables broader access to DNA-free genome editing tools also for laboratories that are not specifically equipped for protein purification.

## 1. Introduction

The cultivation of grapevines, like many other crops, has become increasingly challenging due to shifting climatic conditions and a growing demand for environmentally sustainable agriculture. In this context, New Genomic Techniques (NGTs) are essential. One of the most revolutionary advancements within NGTs is genome editing, which harnesses the formidable potential of the CRISPR-Cas system. This system, originally discovered in bacteria, plays a crucial role in their adaptive immune response [1].

Genome editing with the CRISPR-Cas system precisely targets specific genome locations to introduce single or double cuts to create site-specific mutations that can affect protein functionality by changing amino acids, creating stop codons, or causing frameshift mutations, leading to mutant proteins or gene knockout [2].

The CRISPR-associated proteins (Cas) and guide RNA (gRNA) constitute the ribonucleoproteins (RNPs) of class 2 CRISPR-Cas systems and are the preferred tools for genome editing [3]. Among various Cas proteins, Cas9 and Cas12 are the most prominent due to their unparalleled ability to facilitate precise gene editing through the creation of double-strand breaks (DSBs) in DNA [4]. Cas9 produces blunt-ended DSBs, while Cas12 generates sticky-ended DSBs, following the alignment of gRNAs with specific DNA sequences via complementary base pairing.

To effectively target specific sites and minimize undesirable off-target DNA cleavage, the gRNA must incorporate a specific protospacer adjacent motif (PAM) sequence close to the target site. The versatility in obtaining different PAM sequences empowers the CRISPR-Cas system to reach previously inaccessible targets [5,6].

New Genomic Techniques (NGTs), which enable genetic modifications without introducing exogenous DNA, have emerged as particularly important for high-value species like grapevine, where traditional breeding methods struggle to remove unwanted exogenous DNA sequences. In such cases, utilizing purified RNPs in protoplasts becomes indispensable. NGTs offer the potential to preserve the unique genetic combinations of valuable varieties, ensuring the genome remains unaltered while addressing the specific breeding challenges of these species [7].

Developing new NGTs protocols often requires several attempts, including multiple adjustments to the process’s conditions based on the specific target genotype. This process demands the use of large quantities of RNPs, which are commercially available but can be costly. This financial burden may limit access to the technology for many research groups facing budget constraints, effectively restricting its use primarily to wealthier countries.

Several laboratory protocols for the self-production and purification of RNPs have been published; however, most of these methods require specialized and expensive equipment, making them inaccessible to many researchers.

Lingeman et al. outlined a Cas9 purification protocol where the protein is first passed over a nickel column; then, the His6-maltose binding protein tag is removed by TEV-protease cleavage, followed by intermediate cation exchange column HiTrap SP HP and a final gel filtration step on a Sephacryl S-300 column with the ÄKTA FPLC system [8].

Rajagopalan et al. described a purification protocol for Cas9 divided into two main steps, an affinity enrichment with Ni-NTA resin and then an ion exchange chromatography with the Resource S cation exchange column in an ÄKTA FPLC system [9].

Fleitas et al. published a detailed protocol for the expression and purification of SpCas9, which is fused to GFP and MBP. This method, undertaken in an ÄKTA FPLC system, can yield high quantities of protein, producing up to 30 mg/L of bacterial culture. The GFP fusion is particularly useful for tracking protein delivery; however, the larger size of the protein may pose some challenges. Additionally, the protein must be further processed to remove the MBP before it can be used for in vivo delivery [10].

Flottman et al. outlined a protocol for the expression and purification of recombinant SaCas9 that, like other protocols, utilizes affinity chromatography and size exclusion chromatography with an ÄKTA FPLC system. Notably, cell lysis is accomplished through multiple freeze–thaw cycles of the bacterial pellet; however, the total yield of the protocol was not reported [11].

Lin et al. published a protocol for the purification of Cas9 that requires nickel-affinity chromatography, TEV cleavage, heparin and then gel-filtration chromatography. This protocol is more elaborate compared to the others, but it achieves a reduced endotoxin contamination that is crucial for applications in primary immune cells, which are more sensitive to endotoxin and exogenous RNA [12].

There are fewer published protocols for other Cas proteins besides Cas9. Mohanraju et al. outlined a detailed and efficient protocol for the expression and purification of *Francisella novicida* Cas12a. The purification is divided into two steps; first, the crude lysates are passed through a HisTrap HP column, and then chromatography is performed on a Heparin FF column with the ÄKTA FPLC system [13].

In this work, we present a cost-effective and streamlined protocol for producing various types of RNPs, including the widely used Cas9, a modified Cas12 capable of recognizing altered PAM sites, and a dCas9-VP64 fusion protein designed for gene activation. Our aim is to simplify the production process, enabling the synthesis of active RNPs in laboratories with minimal resources and no specialized equipment for protein purification. By optimizing production steps and reducing dependency on expensive infrastructure, this protocol aims to enhance the accessibility of advanced RNP tools for a wider range of research applications.

## 2. Experimental Design

Several Cas nuclease purification protocols [10,11,12,13,14,15] are based on complex chromatographic methods, such as Fast Protein liquid Chromatography (FPLC), that require specialized equipment. While our protocol produces lower amounts of Cas proteins compared to previously reported methods (maximum 2 mg/L vs. 5–10 mg/L), it offers significant advantages through its simplicity and accessibility. The streamlined workflow is designed to be easily implemented in laboratories with limited instrumentation, removing barriers typically associated with protein purification.

If purified Cas proteins are intended for use in endotoxin-sensitive species, it would be advisable to evaluate endotoxin levels in the preparations. If necessary, additional purification steps, such as the use of endotoxin-removal resins or chromatography techniques specifically designed to bind and reduce endotoxins, could be implemented. These extra measures would help ensure the safety and compatibility of the proteins for sensitive biological systems.

The use of DNA-free editing approaches, such as the direct delivery of RNP complexes, addresses critical challenges associated with traditional plasmid-, viral- or biolistic-based systems. These challenges include the mitigation of off-target effects caused by prolonged nuclease expression [14], the potential genomic instability due to the random integration of exogenous DNA, and regulatory concerns tied to the presence of foreign genetic material. By eliminating the reliance on DNA, RNP-based editing ensures a cleaner and more precise genome modification process, enhancing its appeal for both research and commercial applications, particularly in sensitive species or regulatory environments.

Our protocol advances this approach by simplifying the preparation of active Cas nucleases, making it more accessible to laboratories that lack sophisticated equipment or specialized expertise. This can facilitate the broader application of RNP delivery to cells, significantly reducing the risk of undesired genomic alterations while maintaining good editing efficiency. Furthermore, the flexibility of the protocol allows it to be tailored to accommodate a variety of Cas variants, from traditional Cas9 to specialized forms such as dCas9 for gene regulation or modified Cas12 proteins with expanded PAM recognition.

The protocol’s scalability also ensures that it can be adapted to different experimental needs, whether for small-scale trials or larger projects, while retaining the good purity and activity of the Cas nucleases. This combination of accessibility, flexibility, and reliability positions the protocol as a robust tool for advancing precise genome editing.

By addressing aspects critical to accessibility, this work opens the way for the broader adoption of and innovations in genome editing technologies. Future studies could focus on optimizing expression conditions to enhance nuclease yield and activity.

**Figure 1:**
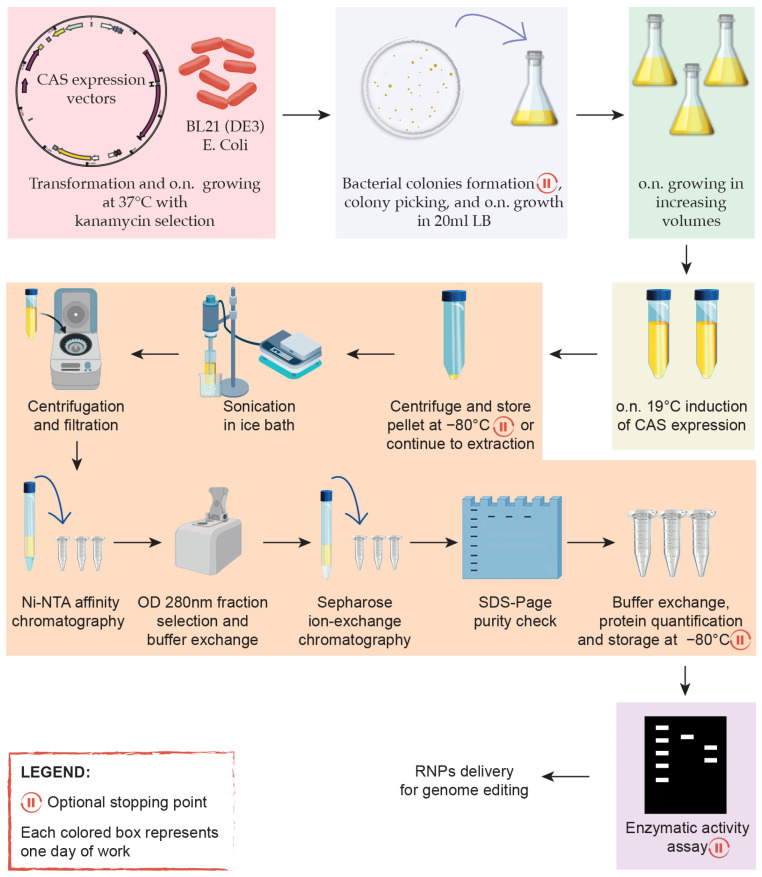
Graphical protocol showing the simplified workflow.

Figure 1 shows the graphical protocol, including the production implementation times and purification stages.

### 2.1. Materials

1.   pET-28b-Cas9-His (Addgene, Cambridge, MA, USA; plasmid no. 47327).
2.   pET-dCas9-VP64-6xHis (Addgene, Cambridge, MA, USA; plasmid no. 62935).
3.   pYPQ230-RR (Addgene, Cambridge, MA, USA; plasmid no. 108860).
4.   Phusion Hot Start II High Fidelity (Thermo Fisher Scientific, Waltham, MA, USA; Cat. no.: F565S).
5.   NcoI (Thermo Fisher Scientific, Waltham, MA, USA; Cat. no.: ER0571).
6.   NotI (Thermo Fisher Scientific, Waltham, MA, USA; Cat. no.: ER0591).
7.   *Escherichia coli* One Shot™ TOP10 (Thermo Fisher Scientific, Waltham, MA, USA; Cat. no.: C404003).
8.   *Escherichia coli* One Shot™ BL21(DE3) (Thermo Fisher Scientific, Waltham, MA, USA; Cat. no.: C600003).
9.   GeneJET Plasmid Miniprep Kit (Thermo Fisher Scientific, Waltham, MA, USA; Cat. no.: K0502).
10. LB Agar (Sigma-Aldrich, St. Louis, MO, USA; Cat. no.: L3147).
11. Kanamycin sulfate (Sigma-Aldrich, St. Louis, MO, USA; Cat. no.: 70560-51-9).
12. IPTG (Sigma-Aldrich, St. Louis, MO, USA; Cat. no.: I5502).
13. HEPES (Sigma-Aldrich, St. Louis, MO, USA; Cat. no.: H3784).
14. Magnesium chloride hexahydrate (Sigma-Aldrich; St. Louis, MO, USA; Cat. no.: 442611).
15. Glycerol (Sigma-Aldrich; St. Louis, MO, USA; Cat. no.: G5516).
16. TCEP (Sigma-Aldrich; St. Louis, MO, USA; Cat. no.: C4706).
17. Lysozyme (Sigma-Aldrich; St. Louis, MO, USA; Cat. no.: L2879).
18. Protease Inhibitor Tablets (Thermo Fisher Scientific, Waltham, MA, USA; Cat. no.: A32963).
19. HisPur Ni-NTA Superflow Agarose (Thermo Fisher Scientific, Waltham, MA, USA; Cat. no.: 25214).
20. Imidazole (Sigma-Aldrich; St. Louis, MO, USA; Cat. no.: I2399).
21. Amicon Ultra-15 centrifugal filters, 30K cutoff (Millipore, Burlington, MA, USA; Cat. no.: UFC503096).
22. SP Sepharose Fast Flow (GE Healthcare; Chicago, IL, USA; Cat. no.: 17072910).
23. Potassium chloride (Sigma-Aldrich; St. Louis, MO, USA; Cat. no.: P3911)
24. DTT (Sigma-Aldrich; St. Louis, MO, USA; Cat. no.: D0632).
25. Bolt™ Bis-Tris Plus Mini Protein Gels, 4–12% (Thermo Fisher Scientific, Waltham, MA, USA; Cat. no.: NW04120BOX).
26. Bolt™ MES SDS Running Buffer 20X (Thermo Fisher Scientific, Waltham, MA, USA; Cat. no.: B0002).
27. NuPAGE™ Sample Reducing Agent 10**×** (Fisher Scientific, Milano, Italia; Cat. no.: 11569166).
28. NuPAGE™ LDS Sample Buffer 4**×** (Fisher Scientific, Milano, Italia; Cat. no.: 11549166).
29. Pierce™ Silver Stain Kit (Thermo Fisher Scientific, Waltham, MA, USA; Cat. no.: 24612).
30. HOT FIREPol DNA Polymerase (Solis BioDyne, Tartu, Estonia; Cat. no.: 01-02-00500).
31. TranscriptAid T7 High Yield Transcription Kit (Thermo Fisher Scientific, Waltham, MA, USA; Cat. no.: K0441).
32. NucleoSpin miRNA kit (Macherey-Nagel, Düren, Nordrhein-Westfalen, Germany; Cat. no.: 740971.50).
33. RNA 6000 Nano Kit (Agilent Technologies, Santa Clara, CA, USA; Cat. no.: 5067-1511).
34. RNAse A 100 mg, resuspended as stock solution at 10 mg/mL and diluted 1:100 for a 0.1 mg/mL working solution (Macherey-Nagel, Düren, Germany; Cat. no.: 740505).
35. Proteinase K (PanReac ApplyChem, Darmstadt, Germany; Cat. no.:A3830).

### 2.2. Equipment

- Thermocycler.
- Standard horizontal gel electrophoresis system.
- Shaking incubator.
- Rotary incubator.
- Refrigerated fixed-angle centrifuge.
- Refrigerated swing-arm centrifuge.
- Sonic Dismembrator Fisherbrand™ Model 120 (Fisher Scientific, Milano, Italy, Cat. no.: 12337338) (or equivalent).
- Nanodrop spectrophotometer (or equivalent).
- (Optional) Mini Gel Tank (Thermo Fisher Scientific, Waltham, MA, USA; Cat. no.: A25977) (or equivalent).
- (Optional) Agilent 2100 Bioanalyzer (Agilent Technologies, Santa Clara, CA, USA) (or equivalent).

## 3. Procedure

This protocol describes the production and purification of three different Cas nucleases expressed from vectors in *E. coli* One Shot BL21(DE3). We used the histidine-tagged Cas9 expression vector pET-28b-Cas9-His (Plasmid #47327), the histidine-tagged, VP64-linked, dCas9 expression vector pET-dCas9-VP64-6xHis (inserted in a pET-29b backbone, plasmid #62935), and a Cas12RR variant fused to an N-terminal His-tag contained in the pYPQ230-RR plasmid (LbCpf1 with G548R and K611R mutations in a Gateway entry plasmid). Two primers (Table S1) were designed to amplify, with Phusion Hot Start II High Fidelity master mix, the Cas12RR gene, introducing NcoI and NotI restriction sites, respectively, at the 5′ and 3′ positions of the transcript. The amplicon was digested with NcoI and NotI and cloned into the pET-28b backbone obtained from the agarose gel separation and purification of a pET-28b-Cas9-His plasmid linearization with the same two enzymes. The obtained pET-28b-Cas12RR plasmid was first cloned in One Shot™ TOP10 Chemically Competent *E. coli* and plated on LB agar with kanamycin (50 µg/mL) for selection, and the chosen colonies were cultured and Sanger sequenced to check for unwanted mutation.

### 3.1. Cas Nucleases Production

The nuclease expression vectors were extracted and purified using a miniprep Plasmid Mini Kit following the manufacturer instructions. *E. coli* One Shot BL21(DE3) competent cells, which allow high levels of expression of recombinant proteins, were transformed with the expression vectors and used for the Cas proteins production. In our protocol, three days are needed to grow enough cells to produce milligrams of Cas proteins starting from single colonies on a plate.

1. Streak transformed E. coli on LB agar plates with kanamycin (50 µg/mL) and incubate at 37 °C overnight.
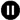
**PAUSE STEP:** plated cells can be stored at 4 °C for up to one week after overnight incubation.
3. Pick a single transformed colony (for each Cas) from the LB plate using a sterile pipette tip to inoculate 20 mL of LB containing kanamycin (50 µg/mL) in a 100 mL flask. Incubate at 37 °C and 160 rpm overnight in a shaking incubator to obtain the starter culture.
4. Inoculate 2 L of LB with kanamycin (50 µg/mL) with 20 mL of starter culture, splitting the culture in five 1 L flasks containing 400 mL each. Incubate at 37 °C in a shaking incubator at 120 rpm until the optical density at 600 nm (OD600) reaches 0.6.
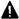
**CRITICAL STEP:** it is necessary to monitor the optical density at 600 nm every hour to prevent it from exceeding the value of 0.6.
5. Incubate the cultures in ice water bath for 20 min to arrest cell growth.
6. Add 0.3 mM filter-sterilized IPTG (Isopropyl-beta-D-thiogalactoside) to each flask to induce expression of Cas proteins in the pETvectors. Incubate the cultures overnight at 19 °C, shaking at 120 rpm.
7. Transfer 400 mL of cultures in 8 × 50 mL tubes and centrifuge at 6000× *g* for 15 min at 4 °C.
8. Removing the supernatant, transfer another 400 mL of cultured cells into the same tubes and repeat the procedure until all cells are harvested. The average yield following this procedure is approximately 8 g of cell pellet.
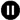
**PAUSE STEP:** Cell pellets could be flash-frozen in liquid nitrogen and stored at −80 °C for some months.

### 3.2. Cas12RR, Cas9 and dCas9-VP64 Extraction

The nucleases were extracted from frozen cell pellets following Rajagopalan et al.’s method with some modification [9].

**Figure 2:**
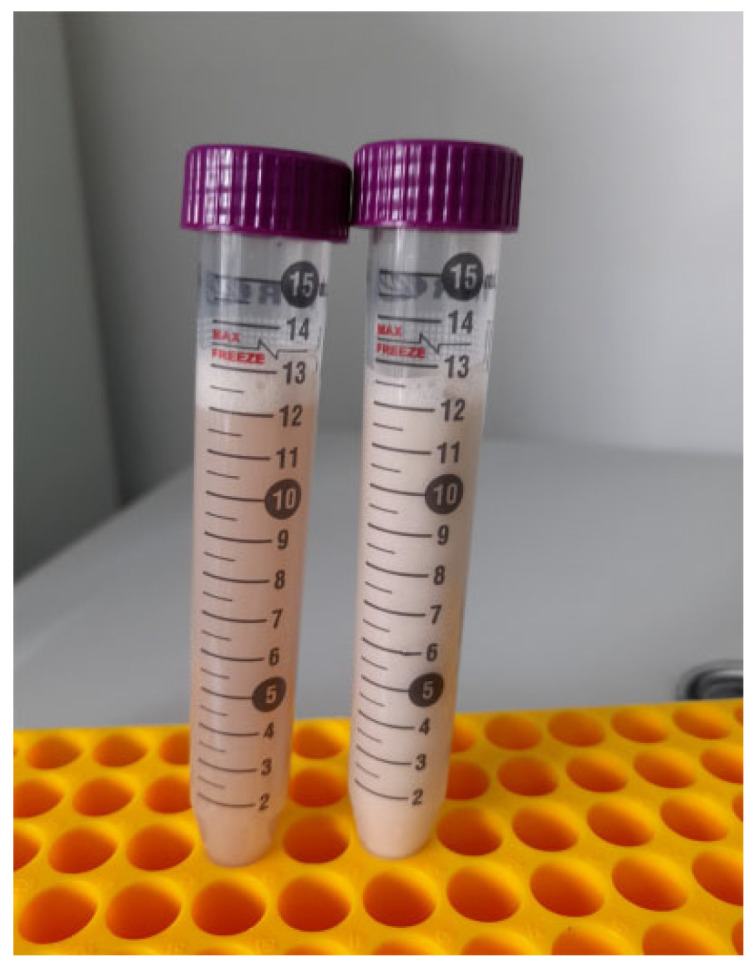
*Escherichia coli* suspension. On the left, after lysis by sonication; on the right, before the lysis.

1. Thaw the cell pellets and suspend them in 40 mL of lysis buffer thoroughly by vortexing.
2. Incubate the cell suspension on ice for 30 min.
3. Sonicate the cell suspension on ice with the Sonic Dismembrator using the following settings: 100% power output, cycles of 5 s ON and 15 s OFF for a total time of 4 min.
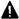
**CRITICAL STEP:** Take great care when sonicating the cell suspension to minimize the increase in sample temperatures, keeping the sample in a 15 mL tube and immersing it in an ice water bath. The cell suspension appears less viscous and brown-tinted after lysis (Figure 2). During the sonication procedure, it is crucial to carefully monitor the temperature of the sample.
4. **OPTIONAL STEP:** Multiple tests may be required to determine the optimal lysis conditions, which depend on factors such as the sample volume, the type of container used, the power and number of cycles employed by the sonicator and the size of the tip. The effectiveness of the cell wall lysis can be assessed by placing a drop of the lysate on a microscope slide, adding 10 microliters of crystal violet (0.05% in 20% methanol) and observing the decrease in intact bacteria under the microscope after each sonication cycle. We have found that the optimal condition to achieve a balance between maximizing lysis and minimizing stress on the proteins is obtained when approximately 10–20% of the bacteria remain intact.
5. Centrifuge the cell lysate at ≥11,000× *g* for 30 min at 4 °C in a swing arm centrifuge to separate the cell debris.
6. Transfer the supernatant into several 2 mL tubes and centrifuge at max RPM (20,000× *g*) for 30 min at 4 °C. This step provides greater clarification of the sample by transitioning from a swing arm centrifuge to a fixed-angle centrifuge. If an ultracentrifuge is available in the lab, perform the previous step at ≥20,000× *g* and skip this step.
7. Filter the supernatant through a 0.22 µm membrane filter and transfer it into a sterile tube.

### 3.3. Nuclease Proteins Purification

The Cas12RR variant, Cas9 and dCas9 VP64 were purified from the filtered lysate under native conditions in two steps: (1) by Ni-NTA affinity chromatography according to Lingeman et al. [8] with some modifications; (2) by Sepharose ion exchange chromatography according to Rajagopalan et al. [9] with some modifications.

#### 3.3.1. Ni-NTA Affinity Chromatography

The His-tagged Cas12RR, Cas9 and dCas9-VP64 were purified from the filtered lysate under native conditions in a bind–wash–elute procedure. The binding of Ni-NTA Agarose with nucleases was performed in batch mode in 15 mL Falcon tubes.

1.   Transfer the HisPur Ni-NTA Superflow Agarose resin (1 mL resin/10 mL lysate) into a 15 mL conical tube and centrifugate at 700× *g* for 1 min to pellet the resin.
2.   Remove and discard the supernatant, then add two resin-bed volumes of deionized water to wash the resin, centrifugate at 700× *g* for 1 min and remove the supernatant; repeat this wash step another time.
3.   Pre-equilibrate the washed resin with five resin-bed volumes of Ni-binding buffer, pipetting gently.
4.   Centrifuge the tubes at 700× *g* for 1 min to remove the Ni-binding buffer supernatant.
5.   Load the filtered lysates onto the resin tubes and mix for 30 min at 4 °C using a rotary shaker at low RPM to avoid foam formation.
6.   Centrifuge the tubes at 700× *g* for 1 min and remove the supernatant.
7.   Wash the resin with two resin-bed volumes of Ni-binding buffer and centrifuge at 700× *g* for 1 min at 4 °C
8.   Carefully remove the supernatant.
9.   Repeat steps 6 and 7 five times.
  **OPTIONAL STEP:** five washes are usually sufficient to eliminate most contaminants that should not bind to the resin. However, if desired, the number of wash repetitions can be increased without significantly affecting protein recovery.
10. Elute bound His-tagged proteins by resuspending the resin, gently pipetting five times in one resin-bed volume of Ni-elution buffer.
11. Incubate the resin on ice for 10 min.
12. Centrifuge the tubes at 700× *g* for 2 min at 4 °C and collect the supernatant.
13. Repeat steps 10–12 at least five more times, collecting a total of six ×1.5 mL fractions. If desired, the number of elutions can be increased.
14. Evaluate the concentration of proteins in the eluted fractions, reading UV absorbance at 280 nm in the Nanodrop spectrophotometer. Fractions with a concentration greater than 0.5 mg/mL are pooled. All our produced nucleases were consistently eluted from the second to the sixth fractions.
15. Concentrate the pooled fractions using centrifugal filters with a 30,000 Da cutoff membrane and centrifuging at 14,000× *g* for 1 min or more at 4 °C.
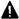
**CRITICAL STEP:** The initial centrifugations of the filters are intended to combine and concentrate the fractions. It is crucial to prevent the drying out of the membranes, which could result in the loss of the sample.
16. Buffer exchange is performed by diluting the concentrated proteins with Ion exchange buffer and centrifugating at 14,000× *g* for 1 min or more at 4 °C in centrifugal filters with a 30,000 Da cutoff membrane. Repeat this step at least five times, collecting a total of approximately 1 mL of final volume.

#### 3.3.2. Sepharose Ion Exchange Chromatography

The Ni-NTA purified Cas nucleases were further purified with SP Sepharose Fast Flow resin performed in batch mode in 15 mL Falcon tubes.

1.   Transfer 2 mL of Sepharose resin into a 15 mL conical tube and wash with 4 mL of deionized water at least twice, then centrifuge at 500× *g* for 1 min to separate the resin from the aqueous phase and allow the easy removal of the supernatant.
2.   Pre-equilibrate the Sepharose resin bed with 8 mL of ion exchange buffer, pipetting gently.
3.   Centrifuge the tubes at 500× *g* for 1 min and remove the ion exchange buffer supernatant.
4.   Load the nucleases from the Section 3.3.1 Ni-NTA purification onto the resin and incubated on ice for 20 min, frequently mixing by inverting the tubes.
5.   **OPTIONAL STEP:** Filter the sample through a 0.22 μm syringe filter before loading onto the Sepharose resin.
6.   Centrifuge the tubes at 500× *g* for 1 min and discard the supernatant.
7.   Wash the resin with four resin-bed volumes of ion exchange buffer by gently mixing.
8.   Centrifuge at 500× *g* for 1 min at 4 °C followed by supernatant removal.
9.   Repeat steps 6 and 7 five times.
10. Elute the Cas nucleases by resuspending the resin, gently pipetting five times in one resin-bed volume of ion exchange buffer with 200 mM KCl.
11. Incubate the resin on ice for 2 min.
12. Centrifuge the tubes at 500× *g* for 2 min at 4 °C and collect the supernatant. Repeat steps 10–12 two more times before proceeding with the elution process from step 10 with a higher concentration of KCl.
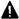
**CRITICAL STEP:** The Cas proteins are eluted by applying stepwise increasing KCl concentrations (200 mM, 300 mM, 400 mM, 500 mM, 600 mM, 700 mM, 800 mM, 900 mM, and 1 M) in the ion exchange buffer. Each salt concentration promotes the release of proteins based on their specific charge characteristics. All of our produced proteins were consistently eluted with the 400 and 500 mM KCl.
13. Repeat steps 10, 11 and 12 three times for each KCl concentration.
14. **OPTIONAL STEP:** Check the presence of Cas nucleases in the eluted fractions by SDS-PAGE.
15. Pool the best supernatants. Concentrate and then buffer exchange five times with the storage buffer using 30,000 Da cutoff centrifugal filters, replicating the procedure previously performed at the end of 3.3.1 for Ni-NTA affinity chromatography.
16. Filter approximately 1 mL of the final volume obtained through a 0.22 µm sterile syringe filter.
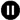
**PAUSE STEP:** Flash-freeze the 100 µL sterile aliquots of proteins with liquid nitrogen and store them at −80 °C. The frozen samples can be stored for several months. While both flash-freezing and −80 °C storage are optional, these are recommended over simple freezing and −20 °C storage, as they will likely extend the shelf life of the active proteins.

### 3.4. Purity Check of RNPs by SDS-PAGE and Absorbances Ratio at 260 and 280 nm

The qualitative control of purified protein content through electrophoresis is technically optional, as it may be considered redundant given the subsequent in vitro enzymatic activity test. However, it is strongly recommended. This step helps identify the potential contamination and unintended degradation of the proteins.

The Cas proteins’ purity was assessed by MES SDS-PAGE in combination with silver staining. MES, MOPS, or standard SDS-PAGE can be used interchangeably; however, MES and MOPS offer faster running times and improved resolution. Zinc staining or Coomassie blue can alternatively be used instead of silver staining; however, the latter offers the highest sensitivity, making it possible to detect even the smallest amounts of contaminant proteins. Each purified nuclease (20 µL) was first prepared by adding LDS (lithium dodecyl sulfate) sample buffer and the reducing agent according to the Novex-life technologies protocol, and then loading this on pre-casted SDS gel with a gradient of 4–12% acrylamide concentration. The SDS-PAGE run was conducted in MES SDS running buffer at 200 V. Immediately after the run, the gel was stained using the Pierce Silver Stain Kit according to the manufacturer’s protocol. The observed sizes of the three Cas proteins we purified were approximately 140 kDa (Cas12RR), 150 kDa (Cas9), and 160 kDa (dCas9-VP64) under our experimental conditions.

Contaminating nucleotides in the produced Cas nucleases can be determined by measuring the 260/280 nm ratio in a spectrophotometer, such as the Nanodrop. The expected ratio for a protein free from nucleic acids ranges between 0.54 and 0.61 [13].

### 3.5. In Vitro gRNA Synthesis

The DNA templates for gRNA targeting regions in exon 1 and exon 7 of the VviAGL11 gene (Vitvi18g02133 in 12X.v2 VCOST.v3 annotation) were amplified by PCR using Hot FirePol and using specific primers listed in Table S1. The amplicons were purified by ethanol precipitation with ammonium acetate. The gRNAs were selected with the Breaking-Cas [15] and CRISPR-P [16] online platform based on their efficiency on the target, particularly when targeting exon 7, where the arginine-197-to-leucine mutation responsible for the seedless phenotype resides [17]. Cas12RR was chosen because, among the PAMs recognized by this nuclease, there is one that allows for cutting exactly across the aforementioned mutation, although with lower efficiency. All gRNAs were transcribed using a T7 promoter. In the case of Cas12 gRNAs, which do not start with a G and therefore would have poor transcription efficiency in vitro with the T7 polymerase, we produced immature pre-gRNAs in high yield, which Cas12 is able to process on its own [18]. The gRNAs were synthesized in vitro using TranscriptAid T7 High Yield Transcription Kit following the manufacturer’s instructions. The reactions were incubated at 37 °C for 8 h and gRNAs were cleaned up with the NucleoSpin miRNA kit.

**OPTIONAL STEP:** The gRNAs’ quality and quantity were assayed by Agilent 2100 Bioanalyzer using Agilent RNA 6000 Nano kit.

### 3.6. In Vitro RNPs Cleavage Assay

The enzymatic activity of CAS nucleases associated to their gRNA was tested using an in vitro assay according to Mohanraju et al.’s protocol [13]. Briefly, a reaction mixture for each Cas protein activity assay was prepared in 1× Cas9 buffer or 1× Cas12 buffer adding Cas nuclease, gRNA and the DNA target at a molar ratio of 1:2:1, respectively, as indicated in Table 1.

The reaction mixture with Cas9 was incubated at 37 °C for 30 min while Cas12RR was incubated for 120 min. Optional step: After the incubation period, 1 µL of 0.1 mg/mL RNAse could be added and incubated at 37 °C for 10 min, then 1 µL of 20 mg/mL proteinase K could be added and incubated at room temperature or at temperatures up to 56 °C for 10 min. The cleavage assays were analyzed on 2% agarose gel.

## 4. Expected Results

The gene encoding Cas12RR, modified to recognize the PAM sequence near the VviAgl11 exon 7 editing site, was successfully cloned into the pET28b expression vector suitable for the high level of expression in the *E. coli* BL21 strain. Induction conditions were optimized for Cas9, dCas9-VP64 and Cas12RR using IPTG concentrations of 0.3 mM at 20 °C overnight to maximize production.

The first step of nucleases purification was carried out through affinity chromatography using Ni-NTA resin exploiting the His-tag present on the nucleases. All proteins were eluted using 250 mM imidazole.

To achieve higher purity, the pooled fractions from Ni-NTA purification were concentrated and buffer-exchanged using a centrifugal filter with a molecular weight cutoff limit of 30,000 and loaded onto an SP Sepharose matrix exploiting the distinct charge properties of these proteins at specific pH values. Following the binding and washing steps, the proteins were eluted with ion exchange buffer with 400 mM and 500 mM KCl, which were the fractions containing most of our Cas proteins.

The elution profiles (Figure S1) showed that Cas9, dCas9-VP64, and Cas12RR were primarily recovered at 400 and 500 mM KCl, indicating the successful separation by the ion exchange approach from other proteins that were washed earlier or were retained in the Sepharose resin.

**Figure 3:**
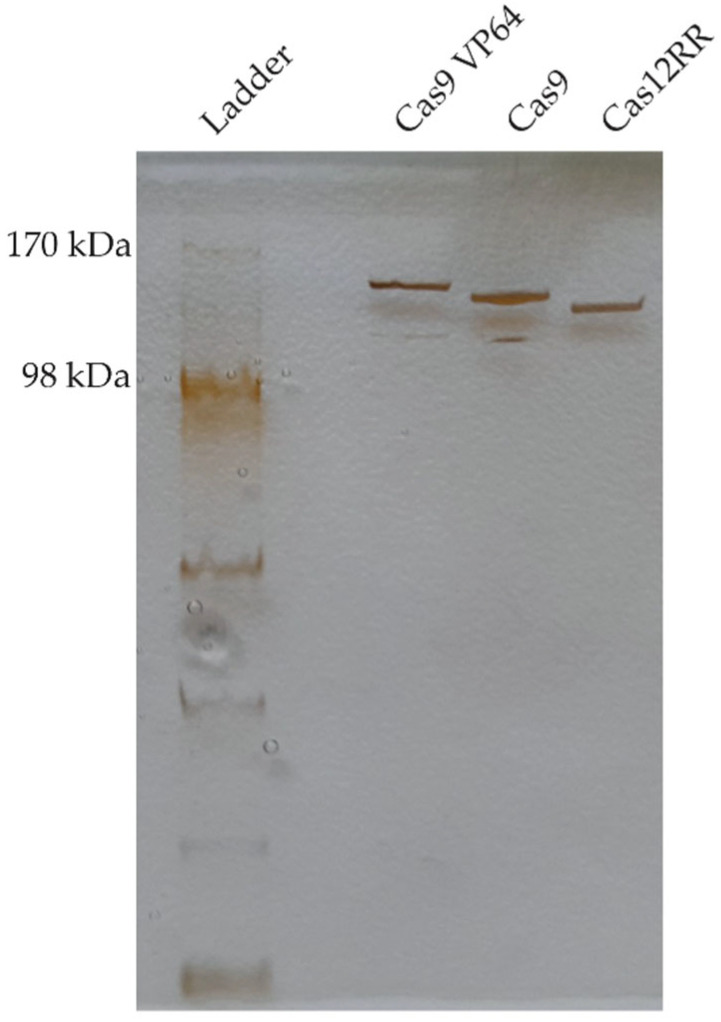
SDS-PAGE analysis of Cas proteins purified on Sepharose ion exchange resin after Ni-NTA affinity chromatography. The illumination of the silver-stained gel causes shadows to appear beneath the strongest bands.

The nucleases purity was assessed by SDS-PAGE. Figure 3 shows single and distinct bands at expected molecular weights of ~140 kDa for Cas12RR, ~150 kDa for Cas9 and ~160 kDa for dCas9-VP64, and with minimal visible impurities, indicating a high level of purity. Purified Cas concentrations were determined by UV absorbance at 280 nm. However, this method can sometimes be imprecise due to the influence of the buffer used or contaminants present. Therefore, it could be beneficial to validate the results with other methods, such as the Bradford assay. The observed 260/280 nm ratios for our Cas proteins were 0.54 for Cas9, 0.54 for Cas12RR, and 0.57 for dCas9-VP64. These values are characteristic of purified proteins that are nucleic acid-free.

On average, this protocol allowed us to produce approximately 1 mg/L, 0.5 mg/L and 2 mg/L of *E. coli* culture for Cas9, dCas9-VP64 and Cas12RR, respectively.

**Figure 4:**
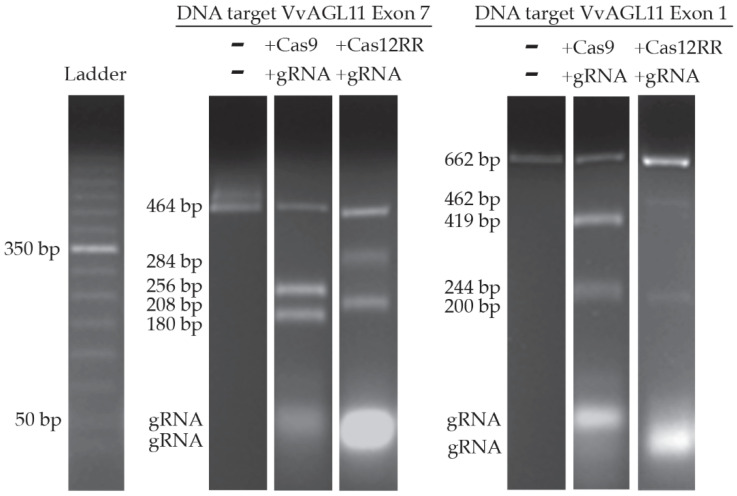
In vitro cleavage test using ribonucleoproteins Cas/gRNA produced and DNA target (VviAGL11-exon1 and VviAgl11-exon7). The image has been cropped solely to enhance clarity.

The enzymatic activities of the purified Cas-gRNAs ribonucleoprotein complex were confirmed by performing in vitro DNA cleavage assays. Cas9 and Cas12RR riboendonucleases successfully cleaved the target VviAGL11 exon 1 (662 bp) and VviAGL11 exon 7 (464 bp) DNA sequences in the presence of their respective guide RNAs, confirming their nuclease activity (Figure 4). When Cas9 was associated to gRNA AGL11_1, it cleaved exon 1, producing the expected bands of 419 bp and 244 bp. Similarly, when Cas9 was associated to gRNA AGL11_7, it cleaved exon 7, producing the expected bands of 256 bp and 208 bp. On the other hand, when Cas12RR was associated to gRNA AGL11_1b, it cleaved exon 1, producing the expected bands of 462 bp and 200 bp. Finally, when Cas12RR was associated to gRNA AGL11_7c, it cleaved exon 7, producing the expected bands of 284 bp and 180 bp.

The cleavage test of the purified proteins was repeated after 2 years of storage at −80 °C and the activity, was conserved.

The functionality of the purified dCAS9-VP64 protein has not been verified in vitro, as this assessment could be challenging. However, since both Cas9 and Cas12RR have here retained their full activity, it is expected that the additional VP64 domain’s activity will be preserved during purification.

In conclusion, the extracted nucleases demonstrate good purity, activity, and stability, ensuring reliable performance in diverse applications.

## 5. Reagents Setup

- **Lysis Buffer.** 20 mM HEPES, 1 mM MgCl_2_, 500 mM NaCl, 10% glycerol, 0.5 mM TCEP, 1 mg/mL lysozyme, 10 mM imidazole, 1 protease inhibitor tablet, pH 7.5. Sterilize through a 0.22 µm sterile syringe filter and store at 4 °C for up to a week.
- **Ni-binding Buffer.** 20 mM Tris-HCl, 500 mM NaCl, 2.5% glycerol, 25 mM imidazole, pH 8. Sterilize through a 0.22 µm sterile syringe filter and store at 4 °C for up to a week.
- **Ni-elution Buffer.** 20 mM Tris-HCl, 500 mM NaCl, 2.5% glycerol, 250 mM imidazole, pH 8. Sterilize through a 0.22 µm sterile syringe filter and store at 4 °C for up to a week.
- **Ion exchange Buffer.** 20 mM HEPES, 200 mM KCl, 5% glycerol, pH 7.5. Sterilize through a 0.22 µm sterile syringe filter and store at 4 °C for up to a week.
- **Storage Buffer.** 20 mM HEPES, 200 mM KCl, 10% glycerol, 1 mM DTT, pH 7.5.
- **10× Cas9 Buffer.** 1 M NaCl, 500 mM Tris-HCl, 100 mM MgCl_2_, 10 mM DTT, pH 7.9. Store at −20 °C for up to one year.
- **10× Cas12RR Buffer.** 1 M NaCl, 200 mM HEPES, 50 mM MgCl2, 1 mM EDTA, pH 6.5. Store at −20 °C for up to one year.

## Figures and Tables

**Table 1:** Ribonucleases activity reaction.

Component	Volume
Nuclease-free water	14 µL
10× Cas9 or Cas12 buffer	2 µL
1 µM gRNA	2 µL
1 µM Cas protein	1 µL
Reaction volume	19 µL
Pre-incubate at room temperature for 20 min	
0.1 µM DNA target (PCR product)	1 µL
Total reaction volume	20 µL

## Data Availability

The original contributions presented in this study are included in the article/Supplementary Material. Further inquiries can be directed to the corresponding author(s).

## Supplementary Materials

The following supporting information can be downloaded at: https://www.mdpi.com/article/10.3390/mps8010016/s1, Table S1: List of primers used in the study. Figure S1: Silver-stained SDS PAGE of Cas9 fractioning with Sepharose Ion exchange resin.
