# Supplementary material for: Simplified Protocol for the Purification of Native Cas Nucleases for DNA-Free Genome Editing
Source: Methods Protoc. 2025 Feb 7;8(1):16. doi: 10.3390/mps8010016 (PMC11857876; doi:10.3390/mps8010016)
Supplement: Supplementary file 1 [file mps-08-00016-s001.zip › Figure S1.pdf]

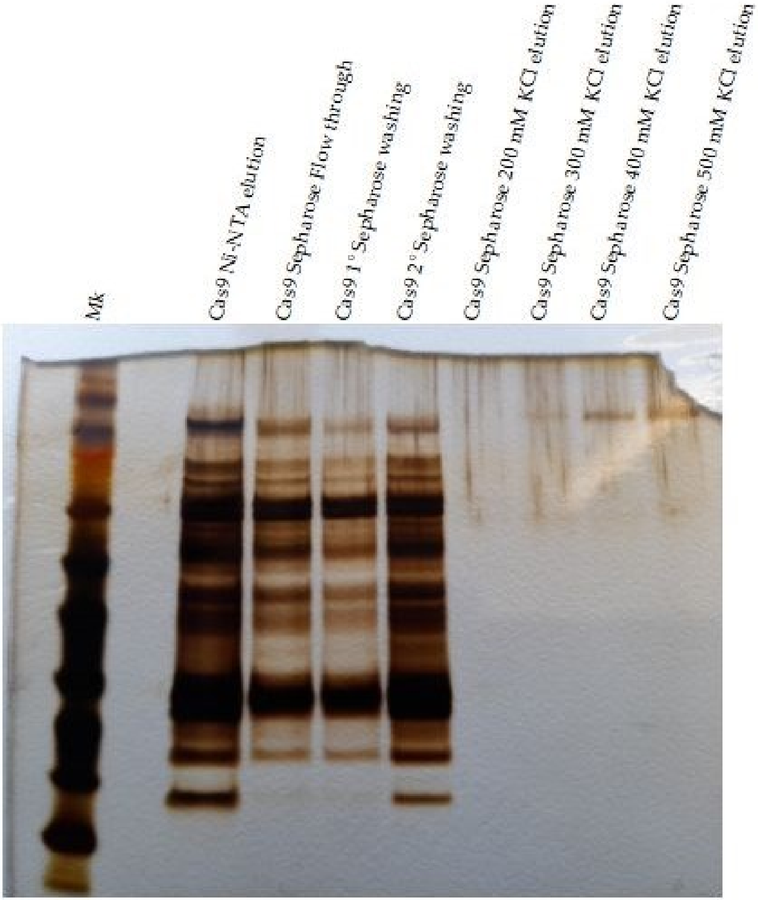

Figure S1: Silver-stained SDS PAGE of Cas9 fractioning with Sepharose Ion exchange resin.

Mk: Molecular weight marker; Ni-NTA eluted Cas9 (1µl); Flow through (5µl); First wash (1µl); Second wash (10µl); 200mM KCl elution (26µl); 300mM KCl elution (26µl); 400mM KCl elution (26µl); 500mM KCl elution (26µl).
